# Extracellular Vesicles in Redox Signaling and Metabolic Regulation in Chronic Kidney Disease

**DOI:** 10.3390/antiox11020356

**Published:** 2022-02-11

**Authors:** Omar Emiliano Aparicio-Trejo, Ana Karina Aranda-Rivera, Horacio Osorio-Alonso, Elena Martínez-Klimova, Laura Gabriela Sánchez-Lozada, José Pedraza-Chaverri, Edilia Tapia

**Affiliations:** 1 Departamento de Fisiopatología Cardio-Renal, Instituto Nacional de Cardiología “Ignacio Chávez”, Mexico City 14080, Mexico; omar.aparicio@cardiologia.org.mx (O.E.A.-T.); horacio.osorio@cardiologia.org.mx (H.O.-A.); laura.sanchez@cardiologia.org.mx (L.G.S.-L.); 2 Laboratorio F-315, Departamento de Biología, Facultad de Química, Universidad Nacional Autónoma de México, Mexico City 04510, Mexico; anitaaranda023@comunidad.unam.mx (A.K.A.-R.); Elena.Martinezklimova@abo.fi (E.M.-K.); pedraza@unam.mx (J.P.-C.)

**Keywords:** extracellular vesicles, exosomes, chronic kidney disease, metabolic reprogramming, redox signaling, microvesicles, mitochondrial impairment, lipotoxicity, oxidative stress, inflammation, fibrosis

## Abstract

Chronic kidney disease (CKD) is a world health problem increasing dramatically. The onset of CKD is driven by several mechanisms; among them, metabolic reprogramming and changes in redox signaling play critical roles in the advancement of inflammation and the subsequent fibrosis, common pathologies observed in all forms of CKD. Extracellular vesicles (EVs) are cell-derived membrane packages strongly associated with cell-cell communication since they transfer several biomolecules that serve as mediators in redox signaling and metabolic reprogramming in the recipient cells. Recent studies suggest that EVs, especially exosomes, the smallest subtype of EVs, play a fundamental role in spreading renal injury in CKD. Therefore, this review summarizes the current information about EVs and their cargos’ participation in metabolic reprogramming and mitochondrial impairment in CKD and their role in redox signaling changes. Finally, we analyze the effects of these EV-induced changes in the amplification of inflammatory and fibrotic processes in the progression of CKD. Furthermore, the data suggest that the identification of the signaling pathways involved in the release of EVs and their cargo under pathological renal conditions can allow the identification of new possible targets of injury spread, with the goal of preventing CKD progression.

## 1. Introduction

Chronic kidney disease (CKD) is a term used to include several disorders characterized by progressive loss in the glomerular filtration rate (GFR) and nephron number for a time period of at least 3 months, usually accompanied by the increase in clinical renal damage markers and fibrotic processes [1,2]. CKD is a global pandemic that is increasing dramatically [3,4,5], and in several cases, the current treatments do not significantly prevent illness [6]. This is partially attributable to the lack of understanding of the several pathological mechanisms that trigger CKD and its progression [6,7].

Extracellular vesicles (EVs) are cell-derived membrane packages released in extracellular medium with a short half-life, from minutes up to 5.5 h after their release. EVs have an essential role in cell-to-cell communication and the maintenance of cellular homeostasis. EVs are currently divided into three groups depending on their origins (biogenesis) and size: exosomes, microvesicles (MVs), and apoptotic bodies [8,9]. The smallest EVs are the exosomes, with a length of 30–150 nm and a density of 1.10–1.18 g/mL [8,10] that are formed by the fusion of intracellular multivesicular bodies, known as endosomes, with the plasma membrane, to be released out of the cell. Microvesicles, 200–1000 nm, are derived from the outward budding of the plasma membrane. On the other hand, the apoptotic bodies, usually >1000 nm, are generated by cell fragmentation induced by apoptosis [9]. Contrary to exosomes and MVs, apoptotic bodies are generated after the disassembly of the cells suffering apoptosis from intracellular fragments. These apoptotic bodies are a hallmark of apoptosis. Intracellular fragments from apoptotic bodies can contain cytosol portions, micronuclei, degraded proteins, chromatin remnants, DNA fragments, or even intact organelles [11]. Little is known about the biological significance of apoptotic bodies over other cells; however, it is shown that lipids, proteins, RNA, and DNA can be found in large-size apoptotic bodies. Moreover, apoptotic bodies are quickly engulfed by macrophages after their release due to their phosphatidylserine groups on display. This mechanism is highly regulated, preventing the activation of inflammation. However, some studies suggest that apoptotic bodies could induce inflammation to promote apoptosis or survival in their neighbor cells [12]. Interesting reviews elegantly address the involvement of apoptotic bodies as signaling molecules in diseases such as cancer [11,13,14].

Exosomes and MVs are widely associated with cell-to-cell communication because they contain and transfer a wide range of biomolecules that can reprogram the recipient cells, such as messenger RNA (mRNA), microRNAs (miRNA), and DNA, as well as proteins and metabolites, including amino acids, lipids, and Krebs cycle intermediates [8,9,10]. It is shown that pathological insults enhance EVs’ release from nephron segments to bodily fluids such as urine and blood, promoting damage, such as fibrosis, which is a common mechanism in the progression of all types of CKD [15,16,17]. Currently, most studies focus on the pathophysiological role of RNAs and proteins included in EVs [18], leaving the metabolites in the EVs poorly studied. EVs secreted by the liver, mesenchymal stem cells, and cancer-associated fibroblasts contain high concentrations of saturated fatty acids (FA), amino acids, and Krebs cycle intermediates. This cargo can reprogram energy metabolism in recipient cells, inhibiting the mitochondrial oxidative phosphorylation system (OXPHOS) and increasing glycolysis [10,18,19]. Additionally, mitochondrial metabolism is tightly regulated by reactive oxygen species (ROS) signaling [20,21], and EVs’ cargo also inhibits mitochondrial metabolism by affecting cellular redox signaling [22,23,24]. These findings could be particularly important for CKD, where growing evidence suggests that metabolic reprogramming and oxidative stress are common mechanisms that affect kidney function, favoring deranged crosstalk between mitochondria and the endoplasmic reticulum (ER), as well as oxidative stress and fibrosis [7,25,26,27]. However, how the EVs’ cargo contributes to energy metabolism reprogramming, oxidative stress, and fibrosis in renal damage progression remains unclear. Therefore, in this review, we summarize the current information about the role of EVs, especially exosomes and MVs, in metabolic and redox impairment and their contribution to CKD development.

## 2. EVs in Kidneys

**Figure 1:**
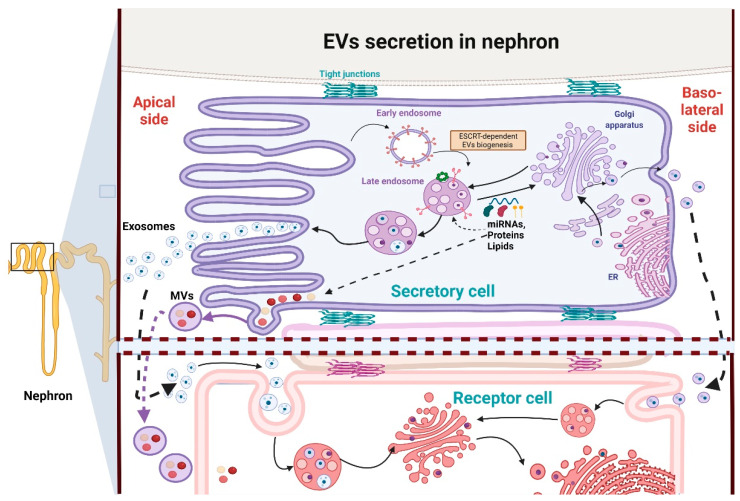
Extracellular vesicles’ (EVs) secretion in nephron segments. In the nephrons, the exosomes and microvesicles (MVs) are strongly affected by the polarity of the cells, especially in a tubular section such as the proximal tubule. Although both the apical and basolateral EVs use components of endosomal sorting complexes required for transport (ESCRT) machinery, the final composition of lipids, proteins, and genetic material is different between them. For example, apical EVs are more enriched in CD24 and lipids such as cardiolipin, ceramide, and phospholipids than basolateral counterparts. Additionally, the apical side releases 3-fold more exosomes under healthy physiological conditions than the basolateral side. EVs allow communication between distant nephron segments since the EVs released from upper nephron segments can be taken in by the cells in lower segments. Image created with Biorender.

Although exosomes and MVs are heterogeneous molecules, they contain some common proteins, most likely from their origin as endosomes, which can be helpful markers for their identification [10]. These proteins are involved in endosomal trafficking such as tetraspanins (CD9, CD63, CD81, etc.), GTPases, annexins and endosomal sorting complexes required for transport (ESCRT), but also heat shock proteins (Hsp90, Hsp60), lipoproteins, and phospholipases [10,28]. It is reported that exosomes contain higher concentrations of cholesterol, sphingolipids, phosphatidylserine, ceramide, and saturated fatty acids than their parent cell [10], which may be related to the fact that EVs are usually released from lipid raft domains in the plasma membrane [9,29]. In fact, the lipid composition of exosomes and lipid rafts is very similar. Therefore, exosomes have higher stability against detergents than other EVs [29]. The formation of these particles depends on the ESCRT machinery (see Figure 1); meanwhile, their secretion relies on both ESCRT and small GTPases [28]. On the other hand, MVs are formed by direct budding from the plasma membrane. [30]. MVs’ formation also recruits the endosomal machinery composed of Ras-related GTPase ADP-ribosylation factor 6 (ARF6), ESCRT machinery, and RHOA-dependent rearrangement of the actin cytoskeleton [31]. These plasma membrane curvatures and rigidity changes imply changes in the protein content, especially aminophospholipid translocases, and lipid components with respect to the parent cell, resulting in an unequal distribution of lipid components in MVs [32]. Although compared to exosomes, the lipid composition of MVs is less known, MVs possess lower concentrations of cholesterol, phosphatidylserine, and other components associated with lipid rafts, making them more sensitive to detergents than exosomes [31,33]. Similar to exosomes, cargo-like proteins and RNA are selectively recruited into MVs, and this selectivity is associated with proteins such as ARF6 and Rab22a (for more detail see [32]). Despite differences in their mechanism of biogenesis and membrane of origin, the two classes of EVs function in similar ways after they are released into the extracellular space [31]. Additionally, EVs’ secretion also relies on the lipid content in lipid rafts such as ceramide because the administration of miRNA and compounds that block the biosynthesis of these lipids inhibits exosome release [10].

The MVs and exosomes contain and transfer a wide range of biomolecules that reprogram the recipient cells in different ways, such as lipids, cytosolic components, active enzymes, histones, cytokines, damage-associated molecular patterns (DAMPs), genetic material such as mRNA, miRNA, and mitochondrial DNA (mtDNA), ligands, and receptors [8,9,10]. However, the content of EVs is not random. The selection of cargo seems to depend on factors such as the cells of origin, external insults such as hypoxia, toxic molecules, physical wounds, and the microenvironment [9,34]. In fact, the cargo can define morphological parameters such as the shape and size of EVs [28]. This cargo can be located inside the EVs or on their membranous surface and help them to adhere to the target cell surface. For example, proteases and glycosidases are found on the surface of the outer layer of the exosomes. This outer layer can present more receptors and membrane proteins than their parent cell [8,9].

Due to the EVs’ participation in cell-to-cell communication, the role of EVs in the genesis and development of renal pathologies is currently under extensive study. Similarly, the use of EVs as biomarkers of renal damage is also currently under extensive study and can be consulted in-depth in several reviews [35]. Therefore, this topic will not be discussed in-depth in this review. Proteomics studies show that exosomes in the nephron originate mainly from kidney resident cells, such as podocytes, proximal convoluted tubules, thick ascending loops of Henle, distal convoluted tubules, and collecting duct cells [9,36,37,38]. Interestingly, exosomes derived from upper nephron segments can be delivered and transfer information to the downstream segments, overpassing even the glomerular filtration barrier [39]. Therefore, the administration of exosomes and other EVs released from healthy cells, especially stem cells, shows beneficial properties in experimental models of CKD and acute kidney injury (AKI), favoring tissue regeneration and kidney repair [8,9]. In contrast, how EVs secreted from injured parenchymal kidney cells promote pathological processes such as fibrosis and inflammation in the recipient kidney cells of lower nephron segments is a topic that is less studied [15,16,17]. Therefore, the role of EVs in the kidneys should not be generalized because it depends on the context of the secretory cells, the physiological context of the recipient cells, the redox and metabolic conditions of both, and the kidneys’ functional state, as well as the type of EVs [16].

In the kidneys, the polarity of the cells strongly affects EVs’ content and secretion. It is reported that the luminal (apical) membrane side releases three-fold more exosomes than the basolateral side (Figure 1), also with a difference in the lipid composition [29]. In this context, urine contains several kinds of exosomes and other EVs, characterized in general by high levels of CD24 and released principally by tubular epithelial and glomerular cells. In contrast, the amount of EVs released into plasma is more limited under non-pathologic conditions [29,34]. The tubular fluid exosomes acts as a tool for the communication between the cells of the nephron since the EVs released from the upper nephron segments can be taken in by the cells in lower segments (Figure 1), so distal tubular and collecting duct cells uptake EVs released by proximal tubules [34]. However, EVs from systemic circulation can also reach tubular epithelial kidney cells [9]. Therefore, blood represents a more systemic measure for EVs’ delivery that also affects kidneys and peripheral organs. Both plasma and urine EVs are particularly important in a pathological context because pathological insults such as hypoxia, nephrotoxicity, proteinuria, and physical wounds trigger the release of EVs from glomeruli and tubular segment cells, promoting the amplification of the injury in the recipient cells [8,15,16]. Therefore, in this review, we first briefly describe the current information on metabolic reprogramming for the promotion of CKD, emphasizing mitochondrial impairment. Next, we summarize the current information about the role of EVs in this metabolic reprograming and in the redox signaling pathways that favor kidney damage progression, with an emphasis on the promotion of inflammatory and fibrotic processes.

### EVs and miRNAs in Kidneys

The EVs contain and transfer a wide range of nucleic acids, among which mRNA, miRNAs, and long noncoding RNAs (lncRNAs) are the most common [40,41,42]. miRNAs are short non-coding, single-stranded RNA molecules with an average of 22 nucleotides in length and have the particularity to be highly conserved sequences between species [41]. miRNAs play a key role in reprogramming the recipient cells through regulation of gene expression via the post-transcriptional processing control of mRNA, transcriptional gene activation, or silencing [41]. These mechanisms of regulation of gene expression modulate metabolic pathways and induce physiological responses, but also activate pathophysiological signaling pathways [41,43]. In fact, recent studies provide evidence that shows crosstalk between miRNAs and components of redox signaling through the modulation of genes related to the formation and removal of ROS [44]. In this context, scientific reports provide evidence that supports the role of miRNAs as potential therapeutic targets in acute and chronic diseases through modulation of signaling pathways, autophagy, mitochondrial metabolism, glycolysis, and redox, among others [45,46,47]. Interestingly, miRNAs contained in EVs are more stable than free miRNAs and are also more specific because they are selectively secreted into the extracellular space and because EV surface proteins can selectively interact with proteins in targets cells [42,48]. There is a big diversity of miRNAs’ cargo in EVs; however, the sorting and loading process of EV-miRNAs is highly selective and is regulated by specific endogenous target sequences. These topics are not the subject of the present work and can be reviewed in recent and beautiful reviews [42,48,49]. Thus, changes in EVs-contained miRNAs’ expression result in impaired cellular function and later lead to metabolic reprogramming and the genesis and development of diseases. More specifically, changes in the miRNAs’ levels are implicated in the pathological processes that lead to the development and progression of CKD; these processes include fibrosis, podocyte damage, inflammation, apoptosis, cell hypertrophy, macrophage proliferation, oxidative stress, and mitochondrial impairment [50,51,52]. In this context, miR-17 is upregulated in experimental models and kidney samples from autosomal dominant polycystic kidney disease patients. MiR-17 inhibits mitochondrial OXPHOS, fatty acid oxidation, and anti-miR-17 attenuated cystic growth by direct repression of peroxisome proliferator-activated receptor alpha (PPARα) [46]. Similarly, miR-21 is upregulated in an experimental model of Alport nephropathy, and miR-21 silencing enhances mitochondrial function and reduces inflammation, glomerulosclerosis, and interstitial fibrosis [53]. Furthermore, MiR-155-5p increased in NRK-52E cell cultures, kidney, and plasma from patients with CKD, and its inhibition reduced oxidative stress and promoted autophagy, thus preventing renal damage [54,55]. Interestingly, these miRNAs are frequently found in exosomes [56,57] Summarizing, because miRNAs regulate gene expression, their role in the progression of CKD is key. Furthermore, miRNAs packaged in EVs represent a pivotal tool for the propagation of the signal, without the degradation that extracellular medium exerts on free miRNAs. In short, the evidence shows the participation of EVs-contained miRNAs in the modulation of signaling pathways associated with lipid, mitochondrial, and glycolytic metabolism, oxidative stress, and autophagy.

## 3. Metabolic Reprogramming in CKD

CKD is developed by a series of acute damages, ischemic episodes, or exposure to nephrotoxic agents, which generate the initial nephron loss. This triggers several pathological mechanisms such as hemodynamic changes, hypertrophy, inflammation, fibrosis, oxidative stress, and metabolic changes [13,27]. The progressive loss of nephrons triggers the hypertrophy of the remnant nephrons, increasing the macromolecular synthesis and the solute reabsorption rates per nephron, which increase the energy consumption in kidneys [13,58,59]. Therefore, this excessive ATP demand in the tubular segment induces stress in ATP sources such as mitochondria [13,14,60,61,62]. Although the mitochondrial changes are dependent on the CKD model cause, there is a consensus in the fact that mitochondria fail to respond to the CKD-induced increase in kidney ATP demand [7,60,63] as shown by the inorganic phosphate accumulation, the decrease in ATP levels, the increase in oxygen uptake, and the reduction in sodium transport in the kidneys [64,65,66,67].

Additionally, the growing accumulation of lipids (lipotoxicity) in kidneys and mitochondrial dysfunction are common pathologies in several types of CKD [2,7,27]. This mitochondrial impairment induces metabolic reprogramming characterized by the shift from mitochondrial-based to anaerobic metabolism, a decrease in β-oxidation, and an increase in ROS production in segments such as proximal tubules, favoring lipotoxicity and CKD progression [68,69]. In this section, we will discuss the energy metabolism change in CKD and the role of EVs-mediated communication in the induction and progression of these changes, with particular attention to mitochondrial impairment and lipotoxicity.

### 3.1. EVs in Metabolic Reprogramming and Mitochondrial Impairment in CKD

Growing evidence suggests that renal mitochondrial impairment triggers several pathological processes that favor CKD progression, such as oxidative stress, Ca^2+^ deregulation, cell death, inflammation, fibrotic processes, and an epithelial-to-mesenchymal transition [7,13,27,70,71,72,73]. In fact, the persistence of mitochondrial impairment in the kidneys over a prolonged time period is fundamental to induce the progression to CKD [62,74,75].

**Figure 2:**
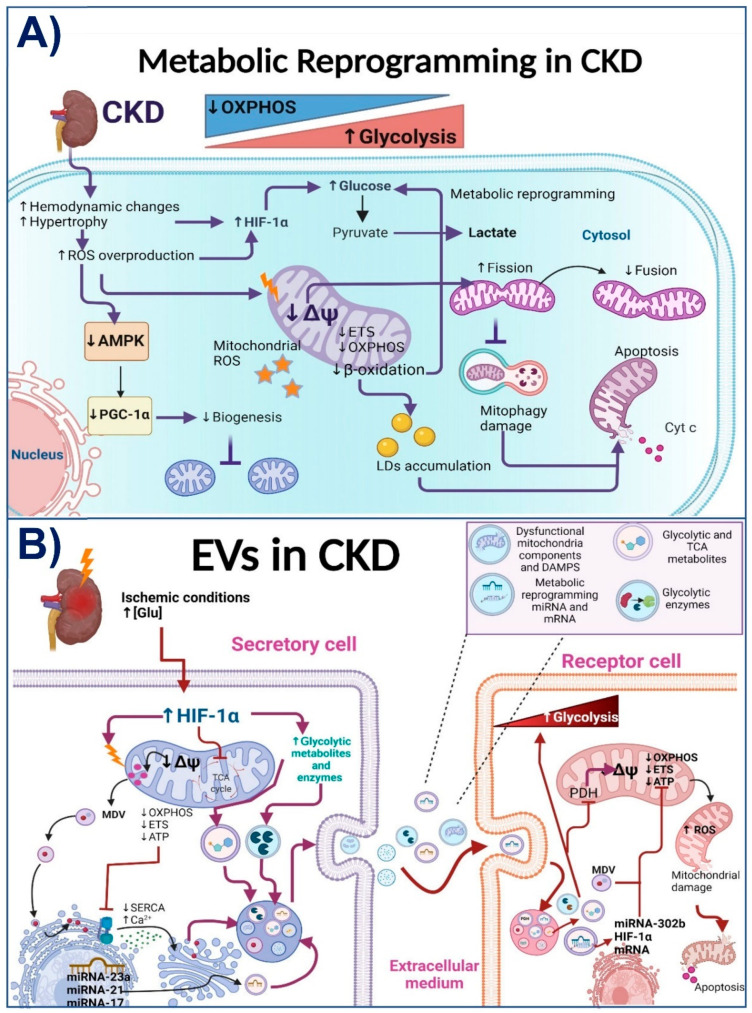
(**A**) Metabolic reprogramming in chronic kidney disease (CKD) in tubular epithelial cells. CKD is characterized by a gradual and progressive shift of energy metabolism from oxidative phosphorylation system (OXPHOS) to glycolysis in the kidney. CKD triggers pathological mechanisms such as hemodynamic changes and hypertrophy. This, together with the reactive oxygen species (ROS) overproduction, favors the activation of hypoxia-inducible factor 1-alpha (HIF-1α), enhancing glycolytic flux and the accumulation of glycolytic metabolites such as pyruvate. Additionally, ROS overproduction decreases protein levels and activation of AMP-activated protein kinase (AMPK) and peroxisome proliferator-activated receptor-gamma coactivator 1-alpha (PGC-1α), reducing mitochondrial biogenesis in the kidney. The mitochondrial impairment induced in CKD is characterized by a decrease in the activity of the electron transport system (ETS) and OXPHOS, which reduces ATP production by decreasing mitochondrial membrane potential (↓ΔΨm). The ΔΨm drop and the oxidative stress in this organelle trigger mitochondrial fragmentation by fission-induction and the subsequent recruitment of the mitophagy machinery. However, the impairment in autophagy flux together with the accumulation of lipid droplets (LDs) derived from a dysfunctional β-oxidation trigger the accumulation of damaged mitochondria; thus, enhancing cellular death and pathological processes. (**B**) Extracellular vehicles (EVs) contribution to metabolic reprogramming in CKD. Ischemic condition or the increase in glucose concentration (↑[Glu]) in CKD triggers the activation of pathological factors such as HIF-1α. This factor promotes mitochondrial impairment by the ΔΨm, OXPHOS, ETS, and ATP production decrease. The ATP level reduction results in sarco-endoplasmic reticulum calcium (Ca^2+^) ATPase (SERCA) activity reduction, increasing Ca^2+^cytosolic levels and the formation and secretion of the EVs. Meanwhile, mitochondrial stress favors the formation of mitochondria-derived vesicles (MDVs), which are loaded with mitochondria-damaged components and tricarboxylic acid (TCA) metabolites under pathological conditions. These MDVs incorporate into multivesicular bodies to be secreted in the form of exosomes. HIF-1α also favors the increase in glycolytic enzymes and metabolites that are incorporated into EVs. In addition, HIF-1α and mitochondrial dysfunction promote the incorporation into EVs of messenger RNA (mRNA) and metabolic reprogramming microRNAs (miRNAs), such as miRNA21, miRNA-23a, and miRNA17. Once internalized, EVs’ cargoes activate the glycolytic pathway and mitochondrial damage in the recipient cell, triggering ROS production and amplifying cell damage to distant nephron segments. PDH: pyruvate dehydrogenase; Cyt c: cytochrome c. Thunder indicates damage. Images created with Biorender.

Subtotal nephrectomy models are extensively used to study the mechanisms involved in CKD genesis and progression [13,76]. In these models, the mitochondrial alteration in kidneys occurs at the early stages of renal tissue loss by the decrease in electron transport system (ETS) activity and mitochondrial coupling [60]. These alterations persist over time and are characterized by a reduction in OXPHOS capacity, mitochondrial membrane potential (ΔΨm) loss, lower complex I and complex III activities, a decrease in bioenergetics mitochondrial protein and mRNA levels, and loss of mitochondrial cristae definition [62,74,77,78,79]. These alterations favor the accumulation of dysfunctional mitochondria and the increase in ROS production (see Figure 2A).

Krebs cycle activity is also dysfunctional in CKD. In kidney biopsies of patients with CKD, there is a reduction in the mRNA levels of Krebs cycle proteins and the urinary excretion of the respective metabolites. This reduction is attributable to the decrease in proteins related to mitochondrial biogenesis, such as AMP-activated protein kinase (AMPK) and peroxisome proliferator-activated receptor-gamma coactivator 1-alpha (PGC-1α) [67,69,80,81,82]. Thus, the decrease in PGC-1α and AMPK is related to the mitochondrial β-oxidation deregulation observed in animal models [14,67,81] and patients [83]. In obesity-related CKD, for example, there is a downregulation of β-oxidation enzymes in the kidneys associated with mitochondrial damage in tubular and glomerular cells [6,26]. Likewise, in diabetes-induced CKD, abnormalities were reported in lipid oxidation [84,85]. This β-oxidation impairment triggers fibrosis development in many segments, especially proximal tubules and glomeruli [62,86]. In fact, mitochondrial β-oxidation impairment is enough to induce the epithelial-mesenchymal transition and transforming growth factor-beta 1 (TGF-β1)-mediated fibrosis in renal cells [69]. This agrees with recent works that suggest that the early mitochondrial ETS impairment triggers the reduction in β-oxidation and mitochondrial ROS overproduction [62,74,75]. Furthermore, fibrosis development in CKD progression is also favored by increasing glycolytic pathways in renal fibroblasts and tubular epithelial cells [87,88]. Together, as we show in Figure 2A, these data suggest that a common mechanism involved in CKD progression is the persistence of mitochondrial bioenergetics alterations. Mitochondrial impairment favors metabolic reprogramming to the anaerobic metabolism and the accumulation of lipids in the renal parenchyma, which then enhances fibrosis in the kidneys.

On the other hand, EVs also carry a variety of metabolites and proteins that regulate the metabolism in recipient cells. Therefore, MVs and exosomes can regulate mitochondrial bioenergetics, biogenesis, redox state, mitophagy, and dynamics in the targets cells [89]. In this way, it is widely characterized that cancer-derived exosomes have the ability to induce metabolic reprogramming in non-cancer cells [19,90]. For example, exosomes secreted by cancer-associated fibroblasts induce the inhibition of ETS, OXPHOS, and oxygen consumption, while at the same time increasing glycolysis and glutamine utilization by the exosome-absorbing cells [19]. Interestingly, high glucose conditions, such as in diabetic nephropathy (DN), can substantially increase EVs’ production with differences in their molecular composition [91]. Furthermore, proteomic studies show that glycolytic enzymes are among the 100 most frequently identified proteins in exosomes and other EVs [92], which suggests that some EVs have a strong ability to induce the prevalence of glycolytic metabolism in their recipient cells [18] (see Figure 2B). The ability of EVs to switch metabolism to glycolysis is associated with hypoxia, which triggers not only changes in the amount of secreted EVs but also in their contents and corresponding functions [89]. For example, hypoxia-derived exosomes contain chemokines, proteins, metabolites, and miRNAs such as miR-21 and miR-17, which promote mitochondrial impairment in recipient cells and subsequent inflammation [53,56,57,89,93]. The exosome-mediated metabolic reprogramming related to hypoxia is not limited to cancer cells [89,94,95]. In fact, in CKD, the hypoxia-inducible factor 1-alpha (HIF1α) is a key regulator of EVs’ production [17,51,95]. HIF-1α promotes glycolytic reprogramming by inducing pyruvate dehydrogenase kinase 1 (PDK1), inhibiting pyruvate dehydrogenase (PDH) and mitochondrial OXPHOS.

Additionally, chronic hypoxia decreases Krebs cycle metabolites such as fumarate and succinate and induces mitochondrial impairment, and the increase in ROS production [51,94,95]. In ischemia/reperfusion and unilateral ureteral obstruction-induced CKD, the HIF-1α increase in tubular epithelial cells induces the enrichment of miRNA-23a in exosomes and their excretion, thus favoring metabolic reprogramming and inflammatory cytokines production in renal macrophages [51]. Inversely, it was also reported that EVs induce the upregulation of miR-302b and HIF-1α levels in their target cells, which also switch energy metabolism towards glycolysis [96], suggesting that these factors can establish a pathological loop. Similarly, exosome-contained miR-21 and miR-17 inhibit mitochondrial OXPHOS by PPARα downregulation, enhancing the glycolytic pathway and tubular and glomerular damage [46,53]. Experimentally, exosomes derived from LLC-PK1 proximal renal tubular cells were loaded with glyceraldehyde-3-phosphate dehydrogenase and delivered to distal convoluted tubules and collecting duct cells, reducing ENaC activity [97], suggesting that the metabolic reprogramming induced by EVs is also propagated to further nephron segments. The importance of exosomes in hypoxia-induced metabolic reprogramming is highlighted by the fact that GW4869 administration, a nSMase2 inhibitor used to suppress exosomes’ secretion, inhibits the glycolytic stimulation in the recipient cells [98].

Exosomes also act as a source of metabolites, such as amino acids, lipids, nucleosides, carbohydrates, and Krebs cycle intermediates, many of which possess functional properties capable of influencing metabolic regulation in recipient cells [18,19,99]. Although up to now, there are very few studies on how exosomes carrying metabolites can facilitate metabolic reprogramming in recipient cells [90], it is known that the change in metabolites’ concentration, induced by pathological stimuli such as hypoxia in CKD, affects the nuclear gene expression of mitochondrial proteins, which is called mitochondrial retrograde signaling [51,95]. Furthermore, researchers found that mitochondrial function and their metabolic composition are affected by the release and uptake of EVs [58]. Although it is still unclear if the metabolites contained in EVs can enter directly into the mitochondria of recipient cells [58], it is well known that EVs can carry damaged and functional mitochondria, mitochondrial proteins, and their metabolites [89,99,100]. This suggests that EVs can carry out active molecules that act on the mitochondria of recipient cells (Figure 2B). In fact, mitochondrial damage triggers the formation of mitochondria-derived vesicles (MDVs) of a size ranging between 70 to 150 nm, as a procedure of mitochondrial quality control to transfer specific mitochondrial particles into the endolysosomal system. This system is also employed for exosome production [51]. However, as we discuss in the next section, the inhibition of degradation pathways such as autophagy enhances the secretion of EVs loaded with damaged mitochondrial components. In contrast, cells under stress conditions can also generate EVs with functional mitochondrial components [19]. For example, mitochondrial membrane proteins are enriched in plasma EVs from patients with melanoma, ovarian cancer, and breast cancer compared to non-cancer-derived EVs. Furthermore, these EVs contain active mitochondrial enzymes [101]. Likewise, EVs derived from brown adipocytes under mitochondrial energy, and oxidative stress are highly enriched in mitochondrial proteins. These EVs increase the rate of oxygen and ADP consumption in recipient cells, suggesting that these enclosed mitochondrial particles have the capacity to stimulate ATP synthesis in their targets [102]. Interestingly, these mitochondrial-associated EVs traverse towards blood circulation and are incorporated into the mitochondrial cardiac network, inducing a transient increase in ROS [102], evidence of mitochondrial transplant in cardiovascular pathologies. Similarly, EVs derived from cancer cells contain miRNAs that target OXPHOS genes, reducing ATP synthase and cytochrome *c* oxidase I transcription, as well as ΔΨm in recipient cells [19].

In contrast, in the stenotic murine kidney model, EVs derived from healthy renal tubular cells are able to restore mitochondrial function on recipient cells by transmitting mitochondria (traveling inside EVs) [103]. Thus, the use of EVs derived from MSCs is currently widely studied as a therapy to reverse mitochondrial impairment in kidney damage models [104,105,106]. This contrasts with the EVs derived from renal cells of CKD models that act as mediators for the dissemination of mitochondrial impairment to different nephron segments [51,96,98]. Briefly, as we show in Figure 2B, injuries such as hypoxia and hyperglycemia can induce the release of EVs from damaged cells in the upper segments of the nephron. The renal secreted EVs are loaded with particles that, once internalized in the recipient cells, impair mitochondrial function, favoring metabolic reprogramming to glycolysis in recipient cells. Furthermore, the EVs-induced mitochondrial damage in recipient cells triggers inflammation, contributing to CKD progression.

### 3.2. EVs in CKD-Associated Lipotoxicity

Lipotoxicity is defined as the accumulation of lipid intermediates in a tissue. Growing evidence suggests that lipotoxicity is a common mechanism in several types of CKD [25,26]. Tubular epithelial cells (TECs) are some of the most energy-demanding cells in the body that usually consume high levels of FAs; however, under pathological conditions, TECs outstandingly accumulate lipids [25,69]. Furthermore, the increase in CD36 levels (the principal system of FAs’ uptake in kidneys) is strongly associated with CKD development [25,107]. CD36 facilitates the uptake of the fatty acids and activates inflammatory and fibrotic pathways [2,62,108]. As later discussed, lipid accumulation also triggers TGF-β activation and macrophage infiltration into the nephrons [109].

On the other hand, it is well known that EVs’ release, specifically exosomes, is increased by lipid accumulation [100]. The role of EVs in lipotoxicity is highlighted by the fact that exosomes are enriched in lipids involved in signaling pathways, proteins, and lipid raft components such as ceramides, cholesterol, sphingomyelin, cardiolipin, lysophosphatidylcholine, prostaglandins, and leukotrienes [29,100,110,111]. Interestingly, it is observed that exosomes derived from cancer cells are enriched in proteins implicated in fatty acid oxidation and in lipid metabolism, which induce metabolic reprograming and an increase in mitochondrial number in targets cells, promoting cancer aggressiveness [100]. In contrast, obese and metabolically unhealthy mice and patients present more blood-circulating EVs with mtDNA and mitochondrial proteins in their damaged form than healthy individuals [102]. In the kidneys, the EVs’ lipid composition depends on the origin of the membrane side. For instance, the exosomes isolated from the basolateral membrane are enriched in cardiolipin, ceramide, and other phospholipids compared to apical-derived exosomes that contain fewer of these lipids, implying differences in their biogenic pathways [110]. However, fatty acids palmitic acid (PA), palmitoleic acid, and oleic acid (OA) are the most abundant lipid species of apical and basolateral plasma membrane-derived exosomes.

**Figure 3:**
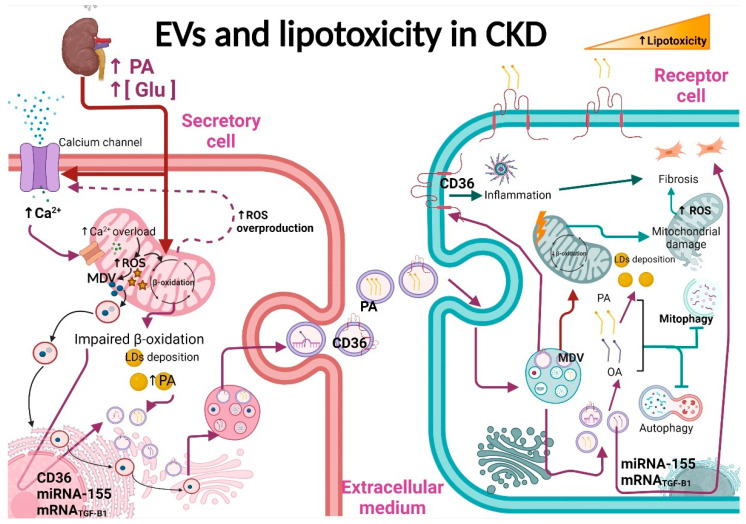
Extracellular vesicles (EVs) and lipotoxicity in CKD. In CKD, the increase in fatty acid levels such as palmitic acid (↑PA) or high glucose (↑[Glu]) concentrations favors the excretion of EVs by activation of the membrane Ca^2+^ (calcium) channels; the cytosolic overload of Ca^2+^ favors the uptake of this ion by the mitochondrial Ca^2+^ uniporter which induces ROS production increase and mitochondrial-derived vesicles’ (MDVs) formation. High levels of PA and ROS induce β-oxidation impairment and, later, accumulation of lipid droplets (LDs) enriched with PA. The LDs’ components and proteins involved in fatty acids’ uptake, such as CD36, are loaded into EVs. Additionally, miRNA-155 and mRNA of transforming growth factor-beta 1 (TGF-β1) are also found in the multivesicular bodies that are secreted as exosomes. Once internalized, EVs release their cargo, such as CD36, which promotes lipid uptake and inflammation in recipient cells. The components of MDVs together with the lipids released in target cells, mainly PA and oleic acid (OA) fatty acids, favor the accumulation of LDs and mitochondrial impairment. Furthermore, PA accumulation also inhibits mitochondria, favoring the accumulation of damaged mitochondria and triggering fibrosis. Image created with Biorender.

In a pathological context, it is reported that EVs secreted after lipid stimulation can trigger inflammatory and fibrotic processes [28]. In rat and human proximal tubular cells, the exposure to PA (16:0) enhanced EVs’ production (Figure 3). Remarkably, apical exosomes contained more palmitoleic acid (16:1) than the basolateral exosomes from the same cells [110]. This increase in exosomes’ release induced by PA was correlated with the increase in apoptosis and the decrease in cell viability of their targets [15]. Interestingly, treatment with a similar concentration of OA, a monounsaturated fatty acid, did not induce EVs’ production, even when OA increased the number of lipid droplets in the secretory cells significantly more than PA [110]. Additionally, PA led to increased lipid accumulation in the ER [15]. Similar results are reported in liver Huh7 cells, where PA enhanced exosome production and increased CD36 expression.

Interestingly, PA altered the miRNAs’ content in exosomes released from hepatic cells without altering the levels of exosome markers such as CD9 and CD63. Furthermore, exosomes from PA-treated hepatocytes had a higher expression of fibrosis markers [111]. Likewise, in PA-treated endothelial cells and high-fat diet-fed mice, the expression of miR-155 levels is increased, positively associated with chronic renal dysfunction, inflammation, and oxidative stress [112]. Remarkably, miR-155 is contained in macrophage-derived exosomes which, when transferred into proximal tubule convoluted cells in vivo and in vitro, enhanced tubular cell senescence [53]. These data suggest that the change in exosomes’ content induced by the increase in fatty acids, especially PA, has an essential role in developing fibrotic processes (Figure 3). In fact, in kidneys, high glucose levels and lipids in DN favor the TGF-β1 increase in glomerular mesangial cells and its release into exosomes, favoring fibrosis in nearby podocyte cells [8]. Additionally, in DN, tubular cells are exposed to higher levels of fatty acids bound to albumin due to damage in the glomerular filtration barrier [15]. The damage in upper nephron segments and basement membrane facilitates that exosomes reach the interstitial space cells, favoring macrophage activation [17]. Together, as we show in Figure 3, these studies suggest that in CKD, the accumulation of fatty acids such as PA induces the secretion of EVs from glomeruli and proximal tubular cells that have pathological effects in recipient cells. As we will discuss in the next section, these EVs are loaded with proinflammatory and profibrotic particles that enhance these processes in recipient cells.

## 4. Redox Signaling and Oxidative Stress in CKD

**Figure 4:**
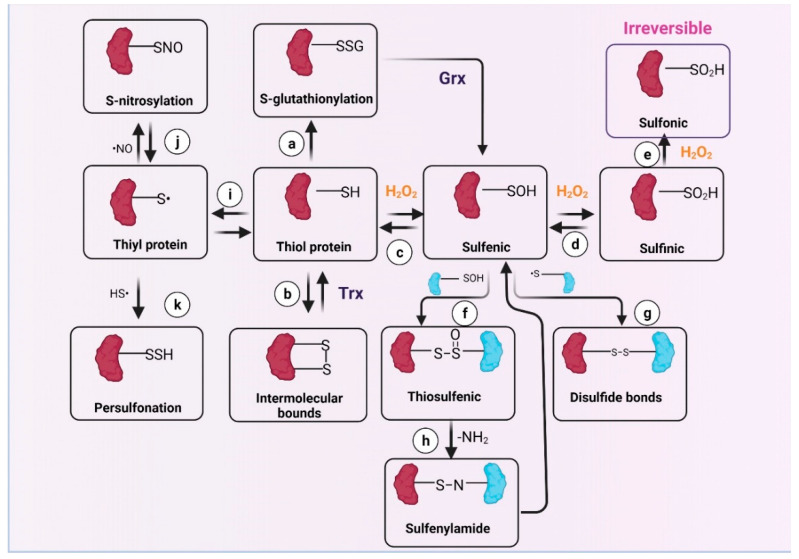
Reactive oxygen species (ROS) induce post-translational modifications in cysteine (Cys) residues that participate in redox signaling. ROS oxidize Cys residues containing thiol groups, forming (**a**) S-glutathionylation (R–SSG), reversed by glutaredoxin (Grx) enzymes. Thiols also can form (**b**) intermolecular bonds, modification reversed by thioredoxin (Trx) system; however, ROS can prompt (**c**) sulfenic form (R–SOH) by suffering oxidation with hydrogen peroxide (H_2_O_2_). If oxidated by H_2_O_2_, R-SOH can give place to (**d**) sulfinic (R–SO_2_H) or (**e**) sulfonic form (R–SO_3_H); the latter is irreversible. Additionally, the oxidation of R–SOH might form (**f**) thiosulfenic [R–S(O)–S-R’] by interacting with another protein containing R-SOH modification or with a protein that contains a thiol group (R–S^•^) to form (**g**) disulfide bonds (R–S–S–R’). R–S(O)–S-R’ might interact with amide (–NH_2_) groups, forming (**h**) sulfenylamide (R–SN–R′), which can be reversed to R–SOH form. On the other hand, an alkaline environment gives rise to deprotonated R-SH protein: (**i**) R–S^•^, which can be modified with nitrosothiol groups (^•^NO), giving rise to (**j**) S-nitrosylation (R-SNO) or can be modified with hydrogen sulfide (H_2_S), forming (**k**) S-persulfonation (R–S–S–H). The red symbols indicate protein 1 that suffers a redox modification in Cys residues, and blue symbols indicate protein 2 that suffers other redox modifications. Image created with Biorender.

Oxidative stress is defined as an imbalance between ROS production and their detoxification by enzymatic and non-enzymatic systems [113], which is well-known to be involved in CKD progression [27,114]. On the other hand, ROS at low concentrations act as second messengers that regulate several signaling pathways in the kidneys [115,116], especially those related to mitochondrial function [117]. At low levels, ROS induce the modification of redox-sensitive protein residues [118], regulating protein function, localization, and stability, depending on the cellular microenvironment [119,120]. The thiol groups of cysteines (Cys) are among the most sensitive redox sensors [21,121,122]. As we show in Figure 4, Cys suffer a wide range of chemical modifications induced by ROS in their thiol group (SH), such as sulfenic acid formation (R-SOH) [123]. R-SOH can react with glutathione (GSH), inducing S-glutathionylation, which prevents subsequent protein oxidation [124]. In addition, R-SOH might conjugate with another R-SOH to form thiosulfinate. These oxidations can be reversed by antioxidant systems such as GSH and thioredoxin, but if oxidation by H_2_O_2_ continues, R-SOH can oxidize to sulfinic acid (R-SO_2_H). Additionally, R-SO_2_H can oxidize to sulfonic acid R-SO_3_H, which is not enzymatically reversible [125]. Other modifications include S-nitrosylation (R-SNO) and persulfide (R–S–SH) formation. As discussed below, these posttranscriptional modifications are mechanisms to interconnect the changes in redox state with the metabolism of mitochondria, which is a fundamental process for the maintenance of homeostasis in the kidneys [126].

Mitochondria and NADPH oxidase (NOX) isoforms 2 and 4 are the main ROS sources in the kidneys and are disturbed during CKD, causing oxidative stress [88]. The oxidative stress leads to inflammation mediated by the activation of nuclear factor kappa-light-chain-enhancer of activated B cells (NF-κB). This mechanism involves the redox modification of Cys residues in IκB, the NF-κB inhibitor, avoiding NF-κB degradation and inducing its translocation to the nucleus. Furthermore, NF-κB also contains redox-sensitive residues in the p50 subunit that suffers glutathionylation, modification required for DNA binding. In models of CKD, the NF-κB protein is upregulated as well as its targets, principally the pro-inflammatory cytokines: tumor necrosis factor-alpha (TNF-α), interleukin 1 beta (IL-1β), and interleukin 6 (IL-6); the adhesion molecules: ICAM and VCAM; E-selectin, chemokines, inducible enzymes such as cyclooxygenase-2 (COX-2), and inducible nitric oxide synthase (iNOS) [127]. The activation of NF-κB is also related to nuclear factor erythroid 2-related factor 2 (Nrf2) downregulation because both transcription factors compete for the transcriptional coactivator cyclic adenosine monophosphate responsive element-binding protein (CREB) [128]. In the CKD context, Nrf2 downregulation favors even more oxidative stress and NF-κB activation [129,130]. Additionally, in DN, the formation of advanced glycation end products (AGES) stimulates NF-κB activation by the mitogen-activated protein kinases (MAPKS) activation pathway. Interestingly, the extracellular signal-regulated kinase (ERK)/Jun N-terminal kinases (JNK) are also activated by some ROS, especially H_2_O_2_, in this model [131]. Indeed, the mechanism involved in the generation of ROS by AGES implies NOX activation, which stimulates the MAPKS pathway [126,132]. This NOX activation also induces TGF-β1 activation, promoting the expression of the fibrotic genes: vimentin, fibronectin, α-smooth muscle actin (α -SMA), and TGF-β1 itself, among others [133]. Summarizing, the impairment in redox signaling generated by mitochondrial and NOX-derived ROS overproduction enhances inflammation and fibrosis in the kidneys.

### 4.1. EVs in Redox Signaling and Oxidative Stress in CKD

Recent studies show that ROS can regulate the release of EVs by the regulatory thiol groups, especially of cell surface-exposed thiols [23,134]. Although the effect of ROS on EVs’ secretion depends on the cell type, the state of the cell, and the proteins with redox-sensitive thiols that the cells express [23], ROS have a strong effect not only on the number of EVs released but also in the composition of the EVs [23,134]. Therefore, in this section, we discuss the principal pathways by which ROS regulate the secretion and the cargo of the EVs and their implications in CKD.

**Figure 5:**
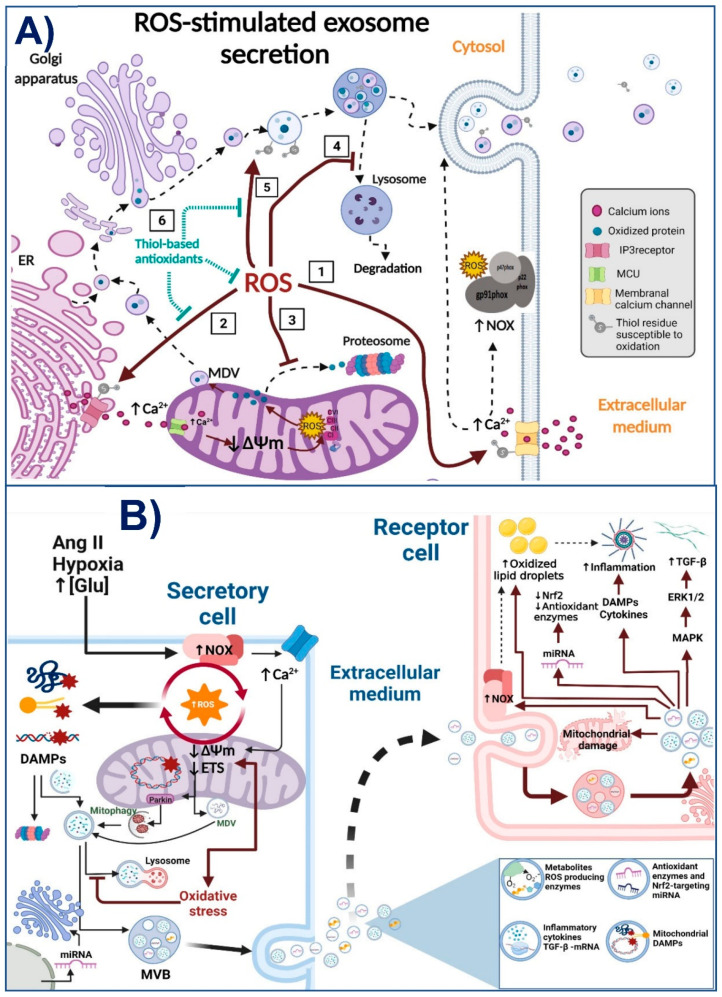
(**A**) Reactive oxygen species (ROS)-induced extracellular vesicles’ (EVs) secretion. ROS induce the EVs’ secretion by (**1**) post-translational modification of cysteine residues (Cys) in the membrane Ca^2+^ channel, regulating the activity of this channel and increasing Ca^2+^ cytosolic concentration. This Ca^2+^ increase can also favor NADPH oxidase (NOX) activation, increasing ROS production even more. Additionally, (**2**) ROS also modulate the inositol trisphosphate receptor (IP3) by Cys modification, increasing the endoplasmic reticulum Ca^2+^ release, which also favors EVs’ release and mitochondrial ROS production by mitochondrial membrane potential (ΔΨm) depolarization and electron transport system (ETS) inhibition. (**3**) ROS inhibit the degradation of damaged mitochondrial components by the proteasome, which enhances the incorporation of these damaged components in mitochondrial-derived vesicles (MDVs). MDVs are normally incorporated in multivesicular bodies (MVBs) to be degraded by autophagy. However, (**4**) under oxidative stress, the inhibition of autophagy flux by ROS prevents the incorporation of the autophagosome into the lysosome, increasing the excretion of mitochondrial damaged components into exosomes. Furthermore, (**5**) ROS can also modify Cys residues of membrane proteins in endosomes, usually proteins involved in lipid rafts; these modifications can affect the traffic of intracellular vesicles and, therefore, exosomes’ secretion. Finally, (**6**) thiol-based antioxidants can regulate the EVs’ content and the EVs’ secretion by thiol redox modification, making them a good tool to study the ROS signaling involved in EVs’ regulation in CKD. (**B**) EVs’ secretion in oxidative stress promotion in CKD. Under pathological stimuli, such as angiotensin II (Ang II), hypoxia or, high glucose [Glu] concentrations, NOX, Ca^2+^, and mitochondria establish a pathological loop of ROS production increase, damaging mitochondrial components such as proteins, lipids, and DNA, thus, favoring the accumulation of damage associated patrons (DAMPs). Under normal conditions, these mitochondrial DAMPs must be degraded by the proteasome or by Parkin-mediated mitophagy of MDVs. However, oxidative stress impairs the degradation pathways, allowing the excretion of these components into the EVs to the extracellular medium. Furthermore, oxidative stress also favors the excretion of exosomes loaded with miRNA against enzymes and antioxidant defense factors such as nuclear factor erythroid 2-related factor 2 (Nrf2). Additionally, ROS-producing enzymes, such as NOX, can be found in their functional form in exosomes. The internalization of all these oxidative stress-promoting components by the recipient cell produces the decrease in antioxidant defense and an increase in ROS production, mitochondrial damage, accumulation of oxidized lipids, DAMPS, and cytokines that trigger the inflammatory response and the activation of the mitogen-activated protein kinases (MAPKS) and transforming growth factor-beta 1 (TGF-β1) pathways in distant segments of the nephron. Image created with Biorender.

One of the key regulators of EVs’ secretion is cytoplasmic Ca^2+^; both MVs and exosome release are strongly affected by the Ca^2+^ cytosolic concentration (see Figure 5A) because it stimulates membrane blebbing and the vesicle fusion with the plasma membrane [135]. It is widely described that many Ca^2+^ channels and proteins involved in Ca^2+^ regulation have redox-sensitive thiol residues (Figure 5A); therefore, they can be activated or inactivated by changes in the ROS concentration [20,99,136,137]. In consequence, the EVs’ release would be promoted or inhibited by ROS, depending on the types of Ca^2+^ channels and Ca^2+^ regulator proteins expressed by the corresponding cell [23]. For example, the transient receptor potential cation channel mucolipin subfamily member 1 (TRPML1) is essential for lysosome trafficking and the lysosome-MVBs’ fusion. In the case of kidneys, it is described that in podocytes, ROS enhance the secretion of inflammatory exosomes by TRPML1 channel activity inhibition, which contributes to glomerular inflammation in hyperhomocysteinemia [138]. Likewise, thiol oxidation of inositol-1,3,5-triphosphate (IP3) receptors by mitochondrial ROS triggers Ca^2+^ flux from the endoplasmic reticulum to the cytoplasm, enhancing EVs’ release and inflammation in recipient cells [139]. Conversely, the Ca^2+^ overload favors the ROS increase. In fact, oxidative stress induced by Ca^2+^ ionophore A23187 stimulates the shedding of EVs in HEK293 cells [140]. Soluble N-ethylmaleimide sensitive factor attachment protein receptors’ (SNAREs) proteins and ATPase N-ethylmaleimide-sensitive factor (NSF) participate in intracellular membrane fusion and EVs’ release. Both NSF and SNAREs are also regulated by thiol modifications [141]. In fact, NSF-induced EVs’ secretion is inhibited by N-ethyl-maleimide (NEM) mediated thiol carbonylation [142], highlighting the role of redox signaling in its activity. Given that the thiol redox state has this regulatory role in EVs’ secretion, EVs’ secretion induction can also be regulated by thiol-based antioxidants (Figure 5A), such as GSH and N-acetylcysteine (NAC) [23]. For example, thiol scavengers 5,5-dithio-bis-(2-nitrobenzoic acid) DTNB and bacitracin can induce EVs’ secretion without affecting cell viability [23]. Furthermore, NAC regulates the composition of exosome cargo [24]. These studies show that the regulation of EVs’ secretion is strongly regulated by the cells’ redox state, and especially by Cys residues.

On the other hand, ROS-induced EVs’ secretion can induce either protective or pathological pathways in target cells, depending on their cargo. For instance, EVs act as scavengers or producers of ROS depending on the physiological or pathological conditions and redox status of the releasing and accepting cells [143]. Thus, ROS are involved in the EVs’ production in secreting cells, but EVs can also induce or detoxify ROS in target cells. Some kinds of EVs can reduce oxidative stress, which is achieved by packaging antioxidant enzyme components in them or by incorporating miRNAs, transcription factors, or other molecules [91]. It is identified that EVs can carry several antioxidant enzymes with functional activity, such as glutathione peroxidase (GPX), glutathione S-transferase (GST), superoxide dismutase (SOD), catalase (CAT), peroxiredoxin (PRX), and thioredoxin (TXN) [91,144,145]. Additionally, recent evidence suggests that EVs can also transfer small molecular weight antioxidants such as NAPDH to protect recipient cells against oxidative stress [102]. For example, functional experiments with EVs derived from endothelial cells show that these EVs could autonomously synthesize NADPH using metabolites present in the blood [146]. Likewise, Nrf2 mRNA and miRNAs involved in the antioxidant response are also packed in exosomes from granulose cells subjected to hydrogen peroxide incubation, thus enhancing Nrf2 signaling and antioxidant enzymes levels of CAT, PRX1, and TXN1 in targets cells [147]. Recently it was found that overexpressing Nrf2 in adipose-derived stem cells induced the secretion of Nrf2-enriched EVs that decrease inflammation and oxidative stress [102]. In cisplatin-induced AKI, miRNA140-5p favors Nrf2 regulation by a mechanism independent of Keap1, promoting the increase in heme oxygenase 1 (HO-1), NAD(P)H quinone oxidoreductase (NQO1), and manganese SOD (MnSOD), which leads to decreasing ROS and attenuating oxidative stress [47]. This miRNA is also contained in exosomes [148]. Similarly, DN miRNA-25 is involved in the downregulation of NOX4 gene expression and oxidative stress [149]. Likewise, miRNA-214 decreased expression in DN is associated with the increase in ROS, lipids, and protein oxidation, as well as the reduction in SOD, GSH, and GSH-PX activity. In contrast, miRNA-214 upregulation significantly decreased oxidative stress, increasing the uncoupling protein 2 (UCP2), p-Akt, and phosphorylated mammalian target of Rapamycin (p-mTOR) [150]. Endothelial cells secreted this miRNA in exomes to prevent senescence [151].

In this context, it must be considered that the vast majority of EVs produced by stem cells have the ability to reduce ROS levels in target cells [143], thus making them a valuable tool for the treatment of CKD, where renal damage is strongly linked to permanent oxidative stress. In a 5/6 nephrectomy model, exosomes from MSC reduce oxidative stress, decreasing kidney fibrosis, interstitial lymphocyte infiltrates, and tubular atrophy [152]. In an ischemia-reperfusion model, MSC-derived exomes improve renal recovery by the miRNAs-mediated downregulation of caspase-3 and SMAD 4, and oxidative stress decrease [153]. Moreover, in ischemia-reperfusion, MSC-derived exosomes attenuated renal dysfunction, histologic damage, and decreased apoptosis by reducing proinflammatory cytokines such as IL-6, TNF-α, NF-kB, and IFN-γ, as well as cleaved-caspase-9 [154]. Interestingly, in acute renal damage, MSC-derived exosomes also inhibited caspase-3 and Bax [154]. Meanwhile, they reduced the levels of 8-hydroxy-2′-deoxyguanosine, malondialdehyde, Bax, and caspase-3 in a *cis*-platinum induced AKI model [155]. Interestingly, protection provided by MSC-derived exosomes against oxidative-stress-induced cell death is also spread to cardiac cells by transferring miR-21 [156], which suggests that these EVs can have peripheral protective effects in CKD-derived pathologies.

In contrast, EVs derived from damaged cells can also deliver oxidized lipids and proteins, triggering detrimental consequences on target cells [145]. This is the case of CKD (Figure 5B). Likewise, Nrf2-targeting miRNA-enriched EVs are secreted in response to cardiac stress. These EVs induce the decrease in Nrf2 signaling in recipient cells and contribute to cardiomyocyte hypertrophy [102]. Surprisingly, all NOX2 subunits required for its activity have also been localized by immune electron microscopy in EVs of around 100 nm, consistent with exosomes’ size. Furthermore, these isolated EVs were able to produce superoxide, and, when are taken in by macrophages and neurons, the result amplified ROS production in these recipient cells [22]. Interestingly, EVs with antagonistic activities, ROS scavengers or ROS producers, may be secreted by the same cells under different stimuli [91,147], confirming that EVs’ cargo depends on the physiological or pathological conditions and redox status of the releasing cells.

In this way, cells exposed to oxidative stress appear to increase the release of EVs [23] with oxidized molecules or pro-oxidant factors such as miRNAs [22,102]. Endothelial cells and fibroblasts exposed to 4-hydroxynonenal (4-HNE) increase the EVs’ release [157], and NOXs and nitric oxide synthase-2 inhibitors can reduce the production of EVs [158]. Interestingly, cardiac cells secrete EVs under stress conditions enriched with various Nrf2-targeting miRNAs that contribute to repressing the Nfr2 signaling pathway favoring pathological alterations [159]. This agrees with the fact that oxidative stress can affect the expression of miRNAs’ regulating genes involved in oxidative stress responses [160]. Similarly, cancer-derived exosomes released under oxidative stress are enriched in transcription factors, RNAs, and oxidized molecules transferred to neighboring or distant cells, modulating their redox status [143]. Furthermore, EVs released under oxidative stress also contain oxidized lipids generated from peroxidation of cell membrane phospholipids, which induce endothelial cell damage and activate neutrophils and monocytes, favoring inflammation [161]. Remarkably, oxidative stress also downregulates the protein concentrations and changes the phosphorylation levels of exosome-contained proteins involved in proliferation and cell survival signaling, as well as energy metabolism [162].

The cells under oxidative stress can transfer via EVs, signaling pathways to spread metabolic changes and cell death in recipient cells [162]. Therefore, it was considered that after oxidative stress, oxidized proteins are usually degraded via the ubiquitin-proteasome pathway or by autophagy. However, when there is an impairment in these pathways, EVs can also serve as an alternative mechanism to remove oxidized proteins by transferring them to other cells, triggering oxidative stress in the last ones [145]. For example, PRX is secreted via EVs; in fact, PRXs contain cysteine residues that need to be oxidized before their inclusion in exosomes; therefore, the inclusion of PRX1 in EVs could be used by cells to eliminate the oxidized PRX protein [163]. This intercellular redox communication via EVs would be fundamental to the progression of several oxidative stress-related diseases, where pro-inflammatory cytokines and immune cell activation are essential for disease development [164,165]. In clinical cases, EVs isolated from the plasma of patients with rheumatoid arthritis or secreted from cells subjected to oxidative stress contained oxidized phospholipids that activate Toll-like receptor 4 (TLR4) [140]. Similarly, aging-related diseases are commonly associated with oxidative stress and inflammation induced by EVs’ release [143]. As we will further discuss, this can be particularly important in CKD, where cell death is promoted along the nephron segments related to oxidative stress [17,51,95]. In target cells, the NF-κB, JNK, and phosphatidylinositol 3-kinase (PI3K)-Akt signaling pathways are usually involved in the pathological changes provoked by ROS-induced EVs [91]. In the kidneys, the ROS increase in podocytes induces the activation of NLR family pyrin domain containing 3 (NLRP3) inflammasomes and the subsequent release of pro-inflammatory exosomes, inducing glomerular inflammation [138]. Similarly, H_2_O_2_ in a dose-dependent manner and NOX activation enhance the formation of EVs in podocytes, giving rise to exosomes containing several inflammatory cytokines [138]. Similarly, the Ang II increase is related to EVs’ release induction mediated by NOX, which stimulated endothelial ROS production and inflammatory responses in recipient cells [166]. Mitochondrial impairment could play a key role in releasing these pro-inflammatory EVs; in fact, the drop in ΔΨm by ROS inhibits mitochondrial Ca^2+^ influx, favoring the increase in the cytosolic Ca^2+^ concentration and EVs’ release [167]. Likewise, the dysfunction in one or several elements of ETS drops ΔΨm, inducing MVs’ and exosomes’ release [156]. A recent study also showed that mitochondrial ROS production, by the perturbation of IP3-receptors and NOX activation, is required by the transient EVs’ production, containing elevated amounts of IL-1β [139]. Furthermore, mitochondrial damage favors the release of mitochondrial content, especially oxidized components (Figure 5B) that act as DAMPs activating an inflammatory response [168]. These components can be packaged and transported by EVs. This topic will be discussed in the next section.

Preventing the oxidative stress in the exosome releasing cells can prevent inflammation in recipient cells. Treatment with CAT or gp91 ds-tat (a NOX inhibitor) in podocytes prevented NLRP3 inflammasome activation in recipient cells [138]. Other EVs can also be employed as a protective preconditioning scheme against oxidative stress. For example, exosomes isolated from granulose cells treated with H_2_O_2_ present high mRNA levels of Nrf2 and downstream antioxidant enzymes such as CAT, PRX1, and TXN1. Once internalized by target cells, these exosomes helped to counteract oxidative stress and mitochondrial damage [147]. Likewise, NAC treatment specifically prevented oxidant-induced detrimental changes in EVs’ signaling without interfering with the physiological functions of EVs [23,24]. A lot of evidence shows that thiol-group-containing antioxidants can prevent pro-inflammatory EVs’ production with anti-oxidative results in target cells [23]. This difference in the EVs’ content between pathological conditions and normal conditions implies the existence of different types of EVs in different redox conditions [91].

In summary, as we show in Figure 5A, ROS regulate the secretion of EVs in the releasing cells via the redox modification of proteins involved in Ca^2+^ regulation, antioxidant systems, and vesicle trafficking, having the Cys and Met residues play a vital role in this regulation. The number of EVs released, as well as their cargo, is also determined by the redox environment of the secretory cells, promoting the packaging of proinflammatory and profibrotic molecules in conditions of oxidative stress. The ability of these ROS-induced EVs to enhance inflammation and fibrosis in CKD, and other renal pathologies, heralds them as mediators in the propagation of the damage along the nephrons induced by oxidative stress (Figure 5B). Therefore, the identification of the signaling pathways involved in EVs’ release and their cargo should allow identifying new possible targets to prevent CKD progression. In this context, selective thiol-containing antioxidants such as NAC and sulforaphane would be excellent tools to prevent pathological changes in EVs without interfering with their normal physiological functions.

### 4.2. EVs-Induced Oxidative Stress and the Promotion of Inflammation and Fibrotic Process in CKD

Inflammation and fibrosis are common mechanisms that support renal damage progression in almost all types of CKD [2,169]. After kidney injury, stimuli such as oxidative stress lead to endothelial and epithelial cells secreting exosomes, chemokines, and cytokines that recruit leukocytes, such as macrophages, lymphocytes, and neutrophils, to the damaged site [170,171]. In this way, kidney fibrosis is characterized by the loss of renal epithelial cells and by replacing the empty spaces with the extracellular matrix [69]. Fibrosis strongly correlates with the deterioration of kidney function, especially oxidative stress [172,173]. Although it is present in several forms of CKD, the mechanisms that induce renal fibrosis are not always the same [174]. In kidneys, hemodynamic alterations, RAS activation, oxidative stress, and Ca^2+^ trigger the activation of maladaptive signaling [2,174,175], principally the TGF-β1 and NF-κB pathways [107,109,176]. The TGF-β1 pathway is considered the main one responsible for developing fibrosis. When it binds to TGF-β1 receptors I and II, pro-fibrotic genes become transcribed by activation of SMAD2, SMAD3, and SMAD4 [177,178]. In this context, the EVs’ release by injured epithelial cells can modulate immune responses by activating both TGF-β1 and NF-κB pathways by transporting DAMPs, cytokines, mtDNA, and miRNAs to the recipient cells [8]. In fact, cytokines and DAMPs packaged and secreted in exosomes are more stable than their free counterparts [8,164]. For example, miRNA-155-5p expression is increased in NRK-52E cell cultures, kidneys from experimental models of DN, and even plasma from patients with CKD [54,55]. MiRNA-155-5 in pathological conditions is found inside of exosomes [179], and its inhibition showed beneficial effects by reducing fibrosis and promoting autophagy by inhibiting the PI3K/Akt/mTOR signaling pathway [54].

**Figure 6:**
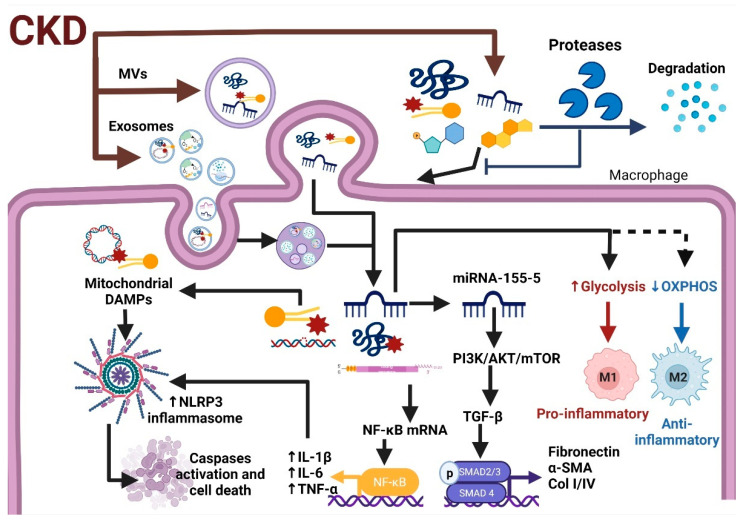
Extracellular vesicles’ (EVs) role in the promotion of inflammation and fibrosis in CKD. Exosomes and microvesicles (MVs) secreted in CKD are enriched with pro-inflammatory factors such as transforming growth factor-beta 1 (TGF-β1) and nuclear factor kappa-light-chain-enhancer of activated B cells (NF-κB). These two factors are the main drivers of inflammation and fibrosis in CKD. Once these EVs are internalized in recipient cells such as macrophages, they increase pro-inflammatory cytokines such as tumor necrosis factor-alpha (TNF-α), interleukin-1 beta (IL-1β), and interleukin 6 (IL-6). Furthermore, mitochondrial DAMPs released by EVs trigger NLR family pyrin domain containing 3 (NLRP3) inflammasome activation and cell death. In addition, miRNA and metabolites released by exosomes induce metabolic reprogramming of macrophages to glycolytic M1 type, the pro-inflammatory type, reducing oxidative phosphorylation system (OXPHOS)-based M2 macrophages, the anti-inflammatory M2 type. Image created with Biorender.

On the other hand, mitochondrial components in EVs can be anti- or pro-inflammatory depending on the context, because oxidized mitochondrial components are more pro-inflammatory than non-oxidized ones [31,90]. It is widely described that some mitochondrial elements act as DAMPs, activating the inflammatory response [168,180]. This can be attributable to differences in the nature of the EVs’ secretory cells and the recipient cells’ conditions. As we previously discussed, the current evidence shows that the content in EVs can also induce changes in cellular metabolism. It is widely described that macrophage activation and the subsequent inflammation are strongly related to changes in metabolic differences between proinflammatory and glycolytic-based metabolism macrophages (M1) and anti-inflammatory and mitochondrial-based metabolism macrophages (M2) [93,181] (see Figure 6). Interestingly, under physiological conditions, EVs containing functional mitochondrial particles increase OXPHOS in the recipient macrophages favoring M2 differentiation [31]. Similarly, macrophages’ endocytosis of mitochondrial EVs released by other cells under basal conditions stimulates their mitochondrial activity and the incorporation of mitochondria from EVs into inflammatory mononuclear phagocytes to restore normal mitochondrial dynamics and cellular metabolism, reducing the expression of pro-inflammatory markers in target cells [182]. Likewise, EVs from MSC or cells under basal conditions serve to modulate the metabolism of distant cells and prevent unwanted immune activation [31].

In contrast, EVs derived from cells under oxidative stress or proinflammatory stimulation, such as exposure to lipopolysaccharides (LPS), are shown to stimulate the production of proinflammatory cytokines [183], which is associated with the impairment in mitochondrial protein quality control systems. For example, in cardiovascular disease, the damage in cardiac macrophages triggers defective elimination of mitochondria, impaired autophagy, and inflammation, and that damage leads to ventricular dysfunction [184]. Under normal conditions, damaged mitochondrial proteins are targeted for lysosomal degradation to prevent the release of these proteins in pro-inflammatory EVs by a process that depends on MDVs. In contrast, pro-inflammatory stimuli such as LPS trigger the release of free mitochondria and EVs loaded with damaged mitochondrial components that stimulate inflammation in recipient cells [89,180]. Consistently with this, the inhibition of lysosomal activity increases the release of mitochondrial EVs that contain oxidatively damaged ETS components and mitochondrial particles, which enter circulation and are taken up by cardiomyocytes, where they trigger a transitory ROS production increase [102]. Interestingly, using ETS inhibitors such as antimycin A in secretory cells, which induce oxidative damage in mitochondria, strongly increased the number of MDVs transported to lysosomes and blocked the inclusion of mitochondrial proteins within EVs [180], as long as the autophagy remained functional. The formation of MDVs and subsequent incorporation of EVs require the recruitment to mitochondria of the Rab9 and Sorting nexin 9 (Snx9) proteins [185]. Interestingly, this recruitment is inhibited by Parkin [180,185], a protein that participates in one of the main mechanisms of mitochondrial protein quality control, the mitophagy [186]. Parkin expression also inhibits the secretion of inner membrane and matrix proteins into EVs instead of sending them to lysosomes for degradation [180]. The mitochondrial fusion-related protein optic atrophy 1 (OPA1) also regulates the formation of the inner membrane and matrix protein charged MDVs, required for the selective inclusion of this mitochondrial content into EVs [180]. Together, these results suggest that cells selectively regulate the incorporation of damaged mitochondrial components within EVs by lysosomal degradation mediated by Parkin-dependent mitophagy to prevent the release of components that would otherwise act as pro-inflammatory DAMPs in recipient cells. This can be particularly important in CKD, where several experimental models [2,27,122,187,188] show that mitophagy and autophagy flux impairment are common pathologies that favor kidney damage progression. In this way, when autophagy flux is impaired, such as in CKD, exosomes and other EVs loaded with oxidized mtDNA and cardiolipin activate the TLR9 pathway and increase pro-inflammatory gene expression [51,89].

Likewise, exosomes can mediate the secretion of NLRP3 inflammasome products out of podocytes, leading to glomerular inflammation and sclerosis [138]. Furthermore, exosomes secreted by macrophages can enhance IL-8 production in renal tubular cells [8]. Thus, the exosomes and other EVs from damaged cells favor macrophage and fibroblast activation, promoting the production of fibrotic molecules such as α-SMA and collagen type I [8,17]. In fact, exosomes released by injured epithelial cells encourage fibroblast proliferation and the expression of fibrosis-associated proteins (Figure 6), such as α-SMA and collagen type I [17]. On the other hand, TGF-β1 administration induces the biogenesis and release of exosomes from renal HKC-8 cells. These exosomes increase fibronectin and collagen deposition in the obstructed kidneys compared to control cell-derived exosomes [36]. In this setting, it is reported that in unilateral ureteral obstruction (UUO), the delivery of TGF-β1 mRNA by exosomes mediated fibroblast activation. In fact, since day 2 of UUO, the obstructed kidney showed higher exosome production than the contralateral non-obstructed kidney, and these exosomes are enriched in TGF-β1 mRNA [17]. The increase in TGF-β1-loaded exosome production was also observed in tubular epithelial cells and glomerular mesangial cells exposed to hypoxic conditions or high glucose, which are also able to activate fibroblasts and promote the spread of injury in recipients cells [8,17]. The importance of exosomes in TGF-β1 pathways is highlighted by the fact that the inhibition of exosomes’ secretion in the UUO model prevented fibrosis and inflammation [17]. In contrast, the activation of repair mechanisms, especially epithelial growth factor (EGF) pathway activation, could reduce the production of exosomes from injured cells. In mouse proximal tubular cells, EGFR inhibition increased exosomes’ production and inhibition of wound healing. Meanwhile, pharmacological inhibitors of exosomes’ secretion such as GW4869 and manumycin A enhance EGFR activation and wound healing [16], suggesting a negative regulation loop between these two factors. Therefore, it is suggested that the exact role of exosomes depends on their source, cell and tissue conditions, microenvironment, and general functional state of the kidneys.

Briefly, in CKD, factors such as oxidative stress, metabolic reprogramming, and the autophagy flux impairment in recipient cells trigger the release of proinflammatory and profibrotic EVs also loaded with mitochondrial associated DAMPs, which are released in the extracellular medium, triggering the activation of immune cells and allowing the spread of the damage to other nephron segments (Figure 6).

## 5. Conclusions

Summarizing, in the kidneys, the secretion of EVs and their content depend on several factors affecting the secretory cells, such as their redox and metabolic status as well as their functional state [17,23,51,91,95,97,134]. Additionally, the polarity of the cells strongly affects EVs’ secretion and their cargo [29]. Thus, the generalization of the EVs’ role in kidneys must be viewed with care. In the context of disease, such as in CKD, the secretion of EVs from injured kidney cells is stimulated by pathological stimuli such as oxidative stress, hypoxia, mitochondrial damage, hyperglycemia, or impairment in autophagy flux [8,15,16,17,51,89,95,152,153,155,168]. These EVs are enriched in miRNA, proteins, transcription factors, functional enzymes, metabolites, and even in whole mitochondria, which, once internalized in the recipient cells, disperse the mitochondrial impairment, favoring metabolic reprogramming to glycolysis and lipid accumulation, and enhance ROS production in the downstream nephron segments. The cell-damaging released EVs and especially exosomes are also loaded with proinflammatory cytokines, mitochondria-associated DAMPs, miRNAs, ROS producer enzymes, transcription factors, and other molecules that favor macrophage differentiation to M1, as well as the promotion of inflammation and fibrosis in recipient cells [8,17,31,36,89,90,180,183], spreading the damage to distant nephron segments, promoting renal impairment in CKD.

In this context, redox signaling mediated by thiol-containing amino acid residues, such as Cys, has a fundamental role not only in mediating the amount of EVs secreted, but also the cargo they contain [23,24,134,138,139,141]. However, more studies are still necessary to elucidate clearly the pathways involved in these redox-mediated mechanisms and to identify possible molecular targets that help prevent the release of EVs loaded with pro-damage cargo without interfering in the role of these vesicles in the normal kidney physiological functions. In this sense, the use of thiol-containing molecules and antioxidants can be useful to prevent the pathological changes in EVs’ release and content [23,24] due to the selectivity in redox signaling that some of them have shown. Finally, the use of MSC-derived EVs has also emerged as a therapeutic alternative to reverse or prevent inflammation, fibrosis, oxidative stress, and even mitochondrial impairment and metabolic reprogramming in renal damage models [31,152,153,154]. Therefore, more studies about the processes involved in the characterization of EVs’ cargo and their internalization processes are still necessary, especially those related to the metabolites contained in EVs and their role in the recipient cells’ reprogramming, given the current lack of this type of studies in CKD models.
